# The gene family-free median of three

**DOI:** 10.1186/s13015-017-0106-z

**Published:** 2017-05-26

**Authors:** Daniel Doerr, Metin Balaban, Pedro Feijão, Cedric Chauve

**Affiliations:** 1 School of Computer and Communication Sciences, EPFL, INJ211 Station 14, 1015 Lausanne, Switzerland; 2 Faculty of Technology and Center for Biotechnology (CeBiTec), Bielefeld University, Universitätsstr. 25, 33615 Bielefeld, Germany; 3 Department of Mathematics, Simon Fraser University, 8888 University Drive, Burnaby, BC V5A 1S6 Canada

**Keywords:** Family-free genome comparison, Positional orthology, Breakpoint median

## Abstract

**Background:**

The gene family-free framework for comparative genomics aims at providing methods for gene order analysis that do not require prior gene family assignment, but work directly on a sequence similarity graph. We study two problems related to the breakpoint median of three genomes, which asks for the construction of a fourth genome that minimizes the sum of breakpoint distances to the input genomes.

**Methods:**

We present a model for constructing a median of three genomes in this family-free setting, based on maximizing an objective function that generalizes the classical breakpoint distance by integrating sequence similarity in the score of a gene adjacency. We study its computational complexity and we describe an integer linear program (ILP) for its exact solution. We further discuss a related problem called *family-free adjacencies for k genomes* for the special case of $k \le 3$ and present an ILP for its solution. However, for this problem, the computation of exact solutions remains intractable for sufficiently large instances. We then proceed to describe a heuristic method, FFAdj-AM, which performs well in practice.

**Results:**

The developed methods compute accurate positional orthologs for genomes comparable in size of bacterial genomes on simulated data and genomic data acquired from the OMA orthology database. In particular, FFAdj-AM performs equally or better when compared to the well-established gene family prediction tool MultiMSOAR.

**Conclusions:**

We study the computational complexity of a new family-free model and present algorithms for its solution. With FFAdj-AM, we propose an appealing alternative to established tools for identifying higher confidence positional orthologs.

**Electronic supplementary material:**

The online version of this article (doi:10.1186/s13015-017-0106-z) contains supplementary material, which is available to authorized users.

## Background

The presented work relates to the branch of research that studies the structural organization of genomes across species. Genome structures are subject to change caused by large-scale mutations. Such mutations permute the order or alter the composition of functional, inheritable entities, subsequently called *genes*, in genome sequences. The *breakpoint median* constitutes a family of well-studied problems that mainly differ through varying karyotypic constraints [1]. A general, unconstrained variant asks to construct a fourth gene order, called a *median*, composed of one or more linear or circular chromosomes, from three given gene orders, such that this median maximizes the sum of conserved gene neighborhoods to the input gene orders. Comparing gene orders of distinct species presupposes knowledge of *positional-* (sometimes also called *main-*) orthologies between their constituting genes. This is where our approach differs from previous work: Whereas traditionally genes are required to form equivalence classes across gene orders such that each genome contains one and only one member of each class, our model only assumes a symmetric and reflexive similarity measure. The tasks of forming one-to-one relationships between genes (i.e. computing a matching) and finding a median are then combined into a single objective. Our approach has the decisive advantage of solving what was formerly a circularity problem: a median provides valuable insights into positional conservation, yet knowledge of positional orthologies are already a prerequisite of traditional breakpoint median problems. Resolving this antilogy, our approach continues a research program outlined in [2] (see also [3]) under the name of *(gene) family-free gene order comparison*. So far, family-free methods have been developed for the pairwise comparison of genomes [4–6] and shown to be effective for orthology analysis [7].

The prediction of evolutionary relationships between genomic sequences is a long-standing problem in computational biology. According to Fitch [8], two genomic sequences are called *homologous* if they descended from a common ancestral sequence. Furthermore, Fitch identifies different events that give rise to a branching point in the phylogeny of homologous sequences, leading to the well-established concepts of orthologous genes (who descend from their last common ancestor through a speciation) and paralogous genes (descending from their last common ancestor through a duplication) [9]. Until quite recently, orthology and paralogy relationships were mostly inferred from sequence similarity. However it is now well accepted that the syntenic context can carry valuable evolutionary information, which has lead to the notion of *positional orthologs* [10], which are orthologs whose syntenic context was not changed in a duplication event.

Most methods for detecting potential orthologous groups require a prior clustering of the genes of the considered genomes into *homologous gene families*, defined as groups of genes assumed to originate from a single ancestral gene. Yet clustering protein sequences into families is already in itself a difficult problem. In the present work, we describe two methods to infer likely positional orthologies for a group of three genomes. The first method solves a new problem we introduce, the *gene family-free median of three*. It generalizes the traditional breakpoint median problem [1]. Our second method makes use of the first exact algorithm that solves the problem *family-free adjacencies for k genomes* (FF-Adjacencies) that has been introduced by Braga  et al. in [2], for the special case where $k \le 3$. We then discuss the methods’ abilities to solve the biological question at hand and study their computational complexity. We show that our approach can be used for positional ortholog prediction in simulated and real data sets of bacterial genomes.

### Related problems

The FF-Median problem relates to previously studied gene order evolution problems. It is a generalization of the tractable mixed multichromosomal median problem introduced in [1], that can indeed be defined as an FF-Median problem with a similarity graph composed of disjoint 3-cliques and edges having all the same weight. The FF-Median problem also bears similarity with problem FF-Adjacencies described in [2] as well as methods aimed at detecting groups of orthologous genes based on gene order evolution, especially the MultiMSOAR [11] algorithm. However, further methods have been proposed that integrate synteny and sequence conservation for inferring orthogroups, see [10]. Our approach differs first and foremost in its family-free principle (all other methods require a prior gene family assignment). Compared to MultiMSOAR, the only other method that can handle more than two genomes with an optimization criterion that considers gene order evolution, both MultiMSOAR (for three genomes) and FF-Median aim at computing a maximum weight tripartite matching. However we differ fundamentally from MultiMSOAR by the full integration of sequence and synteny conservation into the objective function, while MultiMSOAR proceeds first by computing pairwise orthology assignments to define a multipartite graph.

## The gene family-free median of three

### The family-free principle

In the gene family-free framework, we are given all-against-all *gene similarities* through a symmetric and reflexive *similarity measure* $\sigma : \Sigma \times \Sigma \rightarrow \mathbb R_{\ge 0}$ over the universe of genes $\Sigma$ [2]. We use sequence similarity but other similarity measures can fit the previous definition. This leads to the formalization of the *gene similarity graph* [2], i.e. a graph where each vertex corresponds to a gene of the dataset and where each pair of vertices associated with genes of distinct genomes are connected by a strictly positively weighted edge according to gene similarity measure $\sigma$. Then gene family or homology assignments represent a particular subgroup of gene similarity functions that require transitivity. Independent of the particular similarity measure $\sigma$, relations between genes imposed by $\sigma$ are considered as candidates for homology assignments.

#### Extant genomes, genes and adjacencies

In this work, a genome *G* is entirely represented by a tuple $G \equiv (\mathcal C, \mathcal A)$, where $\mathcal C$ denotes a non-empty set of unique genes, and $\mathcal A$ is a set of *adjacencies*. Genes are represented by their *extremities*, i.e., a gene $g \equiv (g^{\text {t}}, g^{\text {h}})$, $g \in \mathcal C$, consists of a *head* $g^{\text {h}}$ and a *tail* $g^{\text {t}}$. Telomeres are modeled explicitly, as special genes of $\mathcal C(G)$ with a single extremity, denoted by “$\circ$”. Extremities $g_1^a, g_2^b$, $a,b \in \{\text {h}, \text {t}\}$ of any two genes $g_1, g_2$ form an *adjacency* $\{g_1^a, g_2^b\}$ if they are immediate neighbors in their genome sequence. In the following, we will conveniently use the notation $\mathcal C(G)$ and $\mathcal A(G)$ to denote the set of genes and the set of adjacencies of genome *G*, respectively. We indicate the presence of an adjacency $\{x^a_1, x_2^b\}$ in an extant genome *X* by

1 $$\begin{aligned} \mathbb I_X(x_1^a, x_2^b)&= {\left\{ \begin{array}{ll}1&{}\text {if } \{x_1^a, x_2^b\} \in \mathcal A(X)\\ 0&{}\text {otherwise.}\end{array}\right. } \end{aligned}$$

Given two genomes *G* and *H* and gene similarity measure $\sigma$, two adjacencies, $\{g_1^a, g_2^b\} \in \mathcal A(G)$ and $\{h_1^a, h_2^b\} \in \mathcal A(H)$ with $a, b \in \{ h, t \}$ are *conserved* iff $\sigma (g_1, h_1) > 0$ and $\sigma (g_2, h_2) > 0$. We subsequently define the *adjacency score* of any four extremities $g^a, h^b, i^c,j^d$, where $a,b,c,d \in \{\text {h, t}\}$ and $g, h, i, j \in \Sigma$ as the geometric mean of their corresponding gene similarities [2]:

2 $$\begin{aligned} s(g^a, h^b, i^c, j^d) \equiv \sqrt{\sigma (g, h) \cdot \sigma (i, j)} \end{aligned}$$

##### Median genome, genes and adjacencies

**Fig. 1:**
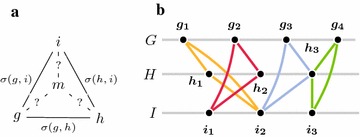
**a** Illustration of the score of a candidate median gene. **b** Gene similarity graph of three genomes *G*, *H*, and *I*. Colored components indicate candidate median genes $m_1 = (g_1, h_1, i_2)$, $m_2 = (g_2, h_2, i_1)$, $m_3 = (g_3, h_3, i_2)$, and $m_4 = (g_4, h_3, i_3)$. Median gene pairs $m_1, m_3$ and $m_3, m_4$ are conflicting

Informally, the family-free median problem asks for a fourth genome *M* that maximizes the sum of pairwise adjacency scores to three given extant genomes *G*, *H*, and *I*. In doing so, the gene content of the requested median *M* must first be defined: each gene $m \in \mathcal C(M)$ must be unambiguously associated with a triple of extant genes (*g*, *h*, *i*), $g \in \mathcal C(G)$, $h \in \mathcal C(H)$, and $i \in \mathcal C(I)$. Moreover, we want to associate to a median gene *m* a sequence similarity score (*g*, *h*, *i*) relative to its extant genes *g*, *h*, and *i*. As the sequence of the median gene is obviously not available, we define this score as the geometric mean of their pairwise similarities (see Fig. 1a):

3 $$\begin{aligned} \sigma (g, m) = \sigma (h, m) = \sigma (i, m) \equiv \root 3 \of {\sigma (g, h) \cdot \sigma (g, i) \cdot \sigma (h, i)} \end{aligned}$$

In the following we make use of mapping $\pi _G(m) \equiv g$, $\pi _H(m) \equiv h$, and $\pi _I(m) \equiv i$ to relate gene *m* with its extant counterparts. Two candidate median genes or telomeres $m_1$ and $m_2$ are *conflicting* if $m_1 \ne m_2$ and the intersection between associated gene sets $\{\pi _G(m_1), \pi _H(m_1), \pi _I(m_1)\}$ and $\{\pi _G( m_2), \pi _H(m_2), \pi _I(m_2)\}$ is non-empty (see Fig. 1b for example). A set of candidate median genes or telomeres $\mathcal C$ is called *conflict-free* if no two of its members $m_1, m_2 \in \mathcal C$ are conflicting. This definition trivially extends to the notion of a *conflict-free* median.

###### **Problem 1**

(*FF-Median*) Given three genomes *G*, *H*, and *I*, and gene similarity measure $\sigma$, find a conflict-free median *M*, which maximizes the following formula:4where $a,b \in \{\text {h}, \text {t}\}$ and $s(\cdot )$ is the adjacency score as defined by Eq. (2).

###### *Remark 1*

The adjacency score for a median adjacency $\{m_1^a, m_2^b\}$ with respect to the corresponding potential extant adjacency $\{\pi _X(m_1)^a, \pi _X(m_2)^b\}$, where $\{m_1^a, m_2^b\} \in \mathcal A(M)$ and $X \in \{G, H, I\}$, can be entirely expressed in terms of pairwise similarities between genes of extant genomes using Eq. (3):

$$\begin{aligned} s(m_1^a, \pi _X(m_1)^a, m_2^b, \pi _X(m_2)^b) = \root 6 \of {\prod _{\{Y, Z\} \subset \{G, H, I\}} \sigma (\pi _Y(m_1), \pi _Z(m_1)) \cdot \sigma (\pi _Y(m_2), \pi _Z(m_2))} \end{aligned}$$

In the following, a median gene *m* and its extant counterparts (*g*, *h*, *i*) are treated as equivalent. We denote the set of all *candidate median genes* by5Each pair of median genes  and extremities $a, b \in \{\text {h, t}\}$ give rise to a *candidate median adjacency* $\{(g_1^a, h_1^a, i_1^a), (g_2^b, h_2^b, i_2^b)\}$ if $(g_1^a, h_1^a, i_1^a) \ne (g_2^b, h_2^b, i_2^b)$, and $(g_1^a, h_1^a, i_1^a)$ and $(g_2^b, h_2^b, i_2^b)$ are non-conflicting. We denote the set of all candidate median adjacencies and the set of all *conserved* (i.e. present in at least one extant genome) candidate median adjacencies by  and , respectively.

###### *Remark 2*

A median gene can only belong to a median adjacency with non-zero adjacency score if all pairwise similarities of its corresponding extant genes *g*, *h*, *i* are non-zero. Thus, the search for median genes can be limited to 3-cliques (triangles) in the tripartite similarity graph.

###### *Remark 3*

The right-hand side of the above formula for the weight of an adjacency is independent of genome *X*. From Eq. (4), an adjacency in median *M* has only an impact in a solution to problem FF-Median if it participates in a gene adjacency in at least one extant genome. So including in a median genome median genes that do not belong to a candidate median adjacency in  do not increase the objective function.

### Accounting for gene family evolution

**Fig. 2:**
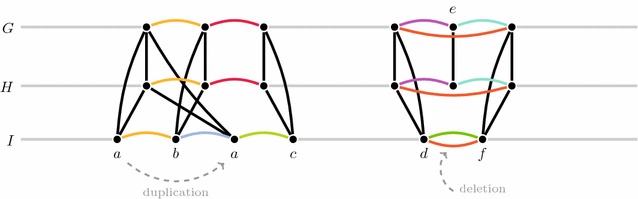
The effect of duplication and deletion of a single gene in problem FF-Median. *Colored arcs* correspond to potential median adjacencies

Duplication and loss are two important phenomena of gene family evolution that affect the gene order. Figure 2 visualizes the outcome of a duplication of a gene belonging to gene family *a* as well as a deletion of a gene from gene family *e*. Both events occurred along the evolutionary path from genome *M* leading to *I*. Such effects of gene family evolution on the gene order must be accounted for in gene order analysis. Yet, they can only be detected once the gene families are inferred. Consequently, family-free methods must provide internal mechanisms for their resolution. Problem FF-Median meets this ambitious demand to some extend. For instance, the true ancestral gene order “*a* *b* *c*” of the example visualized in Fig. 2 will be recovered by solving problem FF-Median as long as the cumulative score of the adjacency between *a* and *b* (yellow arcs), which is conserved in all three extant genomes, plus the score of the twofold conserved adjacency between *b* and *c* (red arcs) is larger than the cumulative score of the onefold conserved adjacencies *b*, *a* (blue arc) and *a*, *c* (green arc) of genome *I*. In other cases where immediate neighborhoods of true positional homologs are less conserved, problem FF-Median likely fails to obtain the correct ancestral gene order. Even worse, it is generally affected by gene deletion events, such as the one shown in the example on the right side of Fig. 2.

In the following, we discuss a related problem called family-free adjacencies, initially introduced by Braga  et al. [2], that can tolerate the effects of both gene duplications and losses.

## Family-free adjacencies for three genomes

In the previous section we introduced problem FF-Median that asks for the construction of a median from three extant genome sequences. In doing so, the median corresponds to a 3-(partite) matching between extant genes that are similar to each other. In this section, we review a more flexible model where the constructed matching also includes smaller components:

### **Definition 1**

(*partial* $\varvec{k}$-*matching*) Given a gene similarity graph $B = (G_1, \ldots , G_k, E)$, a *partial k-matching* $\mathcal M \subseteq E$ is a subset of edges such that for each connected component *C* in $B_{\mathcal M} \equiv (G_1, \ldots , G_k, \mathcal M)$ no two genes in *C* belong to the same genome.

A partial 3-matching $\mathcal M \subseteq E$ in gene similarity graph $B = (G, H, I, E)$ of genomes *G*, *H*, and *I* induces *subgenomes* $G_{\mathcal M} \subseteq G$, $H_{\mathcal M} \subseteq H$, and $I_{\mathcal M} \subseteq I$ with gene sets $\mathcal C(G_{\mathcal M})$, $\mathcal C(H_{\mathcal M})$, and $\mathcal C(I_{\mathcal M})$, respectively, corresponding to the set of vertices incident to edges of matching $\mathcal M$. In doing so, a subgenome $X' \subset X$ may contain adjacencies that are not part of $\mathcal A(X)$: two gene extremities $x_1^a, x_2^b$ form an adjacency $\{x_1^a, x_2^b\} \in \mathcal A(X') \not \subseteq \mathcal A(X)$ iff all genes that lie in between $x_1$ and $x_2$ in genome *X* are not contained in $\mathcal C(X')$.

We then aim to find a partial 3-matching that maximizes a linear combination of a sum of conserved adjacencies and a sum of similarities between the matched genes:

### **Problem 2**

(*family-free adjacencies for three genomes (FF-Adjacencies*) [2]) Given a gene similarity graph $B=(G, H, I, E)$ and some $\alpha$ with $0 \le \alpha \le 1$, find a partial 3-matching $\mathcal M \subseteq E$ that maximizes the following formula:

6 $$\begin{aligned} \mathcal {F}_{\alpha }(\mathcal M)= \alpha \cdot \displaystyle \sum _{\begin{array}{c} \{x_1, y_1\}, \{x_2, y_2\} \in \mathcal M\\ \{x_1^ a , x_2^ b \}, \{y_1^ a , y_2^ b \} \in \mathcal A_{\mathcal M} \end{array}} s(x_1^ a , y_1^ a , x_2^ b , y_2^ b ) \; + \; (1 - \alpha ) \cdot \sum _{(x, y) \in \mathcal M} \sigma (x, y)\,, \end{aligned}$$

where $$\mathcal A_{\mathcal M} = \displaystyle \cup _{X\in \{G, H, I\}} \mathcal A(X_{\mathcal M})$$.

**Fig. 3:**
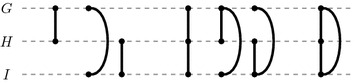
The seven valid types of components of a partial 3-matching

Problem FF-Adjacencies accounts for gene duplications and losses, as well as perturbations in the assessment of gene similarities by (i) considering conserved adjacencies between genes that are not immediate neighbors but lie two, three, or more genes apart, (ii) relaxing the 3-matching to a partial 3-matching, and (iii) maximizing similarities between matched genes. The set of connected components that satisfy the matching constraint form subcomponents of cliques of size three in the gene similarity graph of extant genomes *G*, *H*, and *I*. Figure 3 visualizes the seven possible subcomponents permitted by a partial 3-matching. The matching implies orthology assignments between genes conserved in at least two extant genomes. Because of (iii) and unlike in problem FF-Median, connected components are not bound to participate in conserved adjacencies. Thus, problem FF-Adjacencies can also infer orthology assignments that are unsupported by synteny.

In the next two sections we describe our theoretical results: a study of computational complexity for problems FF-Median and FF-Adjacencies, two methods to compute their exact solutions, and a heuristic that constructs feasible, but possibly suboptimal solutions to FF-Adjacencies based on solutions to problem FF-Median.

## Complexity results

### **Theorem 1**

*Problem FF-Median is MAX SNP-hard*.

We describe the full hardness proof in Additional file 1: Section 1. It is based on a reduction from the Maximum Independent Set for Graphs of Bounded Degree 3. Also, problem FF-Adjacencies has proven NP-hard: Kowada et al. showed that already for the case of pairwise comparisons and uniform similarity scores the problem becomes intractable [6].

In the past decades, numerous problems in the field of computational biology have been shown NP-hard, yet the hope of computing fast solutions has not diminished for all. In fact, many instances of such problems arising in practical applications are less complex and hence can be algorithmically solved rather fast. We are therefore also concerned about the practical computability of the problems at hand. In doing so, we devise methods for computing exact solutions for the comparison of bacterial-sized genomes in the next section. We present FF-Median, an integer linear program (ILP), for the solution of the correspondent problem. In order to speed up the computation in practice, we additionally present algorithm ICF-SEG that detects local optimal structures that commonly appear when comparing genomes of reasonably close species.

Further, we present ILP FFAdj-3G for the solution of problem FF-Adjacencies. However, the problem’s superior capability (compared to problem FF-Median) of resolving events of gene family evolution comes at the expense of a dramatically increased search space. Taking adjacencies between genes into consideration that are further apart leads to an explosion of conflicting conserved adjacencies. This number is then potentized by the number of possible subcomponents in a partial 3-matching, making the computation of solutions even more challenging. Thus, it is impossible to calculate exact solutions to problem FF-Adjacencies with program FFAdj-3G for average-sized bacterial genomes in reasonable runtime. Addressing problem FF-Adjacencies in pairwise comparisons, Doerr proposed in [3] an effective method to identify optimal substructures in practical instances, allowing the computation of exact solutions for bacterial-sized genomes. As of the time of writing, the search for similar structures in the case of three genomes has been unsuccessful. Therefore, we propose an alternative, practically motivated method, called FFAdj-AM, which first computes a solution to problem FF-Median, then treating the matching implied by the obtained median as invariant in the search for a (possibly suboptimal) solution to problem FF-Adjacencies. (Note that every solution to FF-Median is a feasible solution to problem FF-Adjacencies.) More precisely, FFAdj-AM calls first program FF-Median on a given gene similarity graph $B = (G, H, I, E)$ and subsequently treats its output as a partial, feasible solution for problem FF-Adjacencies. Then it executes program FFAdj-3G to improve on this solution by exploring the subgraph of *B* that is not contained in the initially computed family-free median. This approach turns out to be feasible in practice. We show this in our evaluation by computing exact solutions on a biological dataset composed of 15 $\gamma$-proteobacterial genomes.

## Algorithmic results

### An exact ILP algorithm to problem FF-Median

**Fig. 4:**
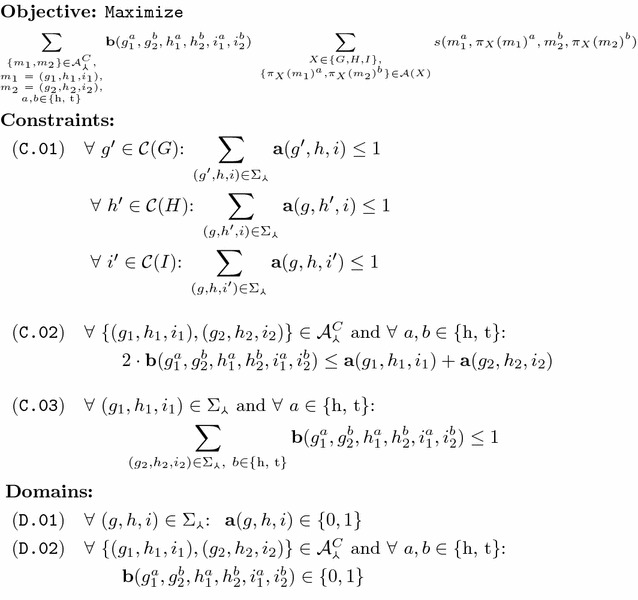
Program FF-Median, an ILP for solving problem FF-Median

We now present program FF-Median, described in Fig. 4, that exploits the specific properties of problem FF-Median to design an ILP using $\mathcal O(n^5)$ variables and statements. Program FF-Median makes use of two types of binary variables $\mathbf a$ and $\mathbf b$ as declared in domain specifications (D.01) and (D.02), that defines the set of median genes  and of candidate conserved median adjacencies  (Remark 3). The former variable type indicates the presence or absence of candidate genes in an optimal median *M*. The latter, variable type $\mathbf b$, specifies if an adjacency between two gene extremities or telomeres is established in *M*. Constraint (C.01) ensures that *M* is conflict-free, by demanding that each extant gene (or telomere) can be associated with at most one median gene (or telomere). Further, constraint (C.02) dictates that a median adjacency can only be established between genes that both are part of the median. Lastly, constraint (C.03) guarantees that each gene extremity and telomere of the median participates in at most one adjacency.

#### **Property 1**

*The size (i.e. number of variables and statements) of any ILP returned by program* FF-Median *is limited by* $\mathcal O(n^5)$ *where* $n=\max (|\mathcal C(G)|,|\mathcal C(H)|,|\mathcal C(I)|)$.

#### *Remark 4*

The output of the algorithm FF-Median is a set of adjacencies between median genes that define a set of linear and/or circular orders, called CARs (Contiguous Ancestral Regions), where linear segments are not capped by telomeres. So formally the computed median might not be a valid genome. However, as adding adjacencies that do not belong to  do not modify the score of a given median, a set of median adjacencies can always be completed into a valid genome by such adjacencies that join the linear segments together and add telomeres. These extra adjacencies would not be supported by any extant genome and thus can be considered as dubious, and in our implementation, we only return the median adjacencies computed by the ILP, i.e. a subset of .

#### *Remark 5*

Following Remark 2, preprocessing the input extant genomes requires to handle the extant genes that do not belong to at least one 3-clique in the similarity graph. Such genes can not be part of any median. So one could decide to leave them in the input, and the ILP can handle them and ensures they are never part of the output solution. However, discarding them from the extant genomes can help recover adjacencies that have been disrupted by the insertion of a mobile element for example, so in our implementation we follow this approach.

As discussed at the end of the previous section, the FF-Median problem is a generalization of the mixed multichromosomal breakpoint median problem [1]. Tannier et al. presented in [1] an approach for its solution based on a Maximum-Weight Matching (MWM) algorithm. This motivates the results presented in the next paragraph that also use a MWM algorithm to identify optimal median substructures by focusing on conflict-free sets of median genes.

#### Finding local optimal segments

Tannier et al. [1] solve the mixed multichromosomal breakpoint median problem by transforming it into an MWM problem, that we outline now. A graph is defined in which each extremity of a candidate median gene and each telomere gives rise to a vertex. Any two vertices are connected by an edge, weighted according to the number of observed adjacencies between the two gene extremities in extant genomes. Edges corresponding to adjacencies between a gene extremity and telomeres are weighted only by half as much. An MWM in this graph induces a set of adjacencies that defines an optimal median.

We first describe how this approach applies to our problem. We define a graph  constructed from an FF-Median instance $(G, H, I, \sigma )$ that is similar to that of Tannier et al. deviating by defining vertices as candidate median gene extremities and weighting an edge between two vertices $m_1^a, m_2^b$, $a, b \in \{ h, t \}$, by

7 $$\begin{aligned} {\begin{matrix} w(\{m_1^a, m_2^b\})&= \displaystyle \sum _{X \in \{G, H, I\}} \mathbb I_X(\pi _X(m_1)^a, \pi _X(m_2)^b) \cdot s(m_1^a, \pi _X(m_1)^a, m_2^b, \pi _X(m_2)^b). \end{matrix}} \end{aligned}$$

We make first the following observation, where a conflict-free matching is a matching that does not contain two conflicting vertices (candidate median genes):

##### **Observation 1**

*Any conflict-free matching in graph*  *of maximum weight defines an optimal median*.

We show now that we can define notions of sub-instances—of a full FF-Median instance—that contains no internal conflicts, for which applying the MWM can allow to detect if the set of median genes defining the sub-instance is part of at least one optimal FF-Median. Let $\mathcal S$ be a set of candidate median genes. An *internal conflict* is a conflict between two genes from $\mathcal S$; an *external conflict* is a conflict between a gene from $\mathcal S$ and a candidate median gene not in $\mathcal S$. We say that $\mathcal S$ is *contiguous* in extant genome *X* if the set $\pi _X({\mathcal S})$ forms a unique, contiguous, segment in *X*. We say that $\mathcal S$ is an *internal conflict-free segment* (IC-free segment) if it contains no internal conflict and is contiguous in all three extant genomes; this can be seen as the family-free equivalent of the notion of *common interval in permutations* [12]. An IC-free segment is a *run* if the order of the extant genes is conserved in all three extant genomes, up to a full reversal of the segment.

Intuitively, one can find an optimal solution to the sub-instance defined by an IC-free segment, but it might not be part of an optimal median for the whole instance due to side effects of the rest of the instance. So we need to adapt the graph to which we apply an MWM algorithm to account for such side effects. To do so, we define the *potential* of a candidate median gene *m* as We then extend graph $\Gamma ( \mathcal S) =: (V, E)$ to graph $\Gamma '( \mathcal S) := (V, E')$ by adding edges between the extremities of each candidate median gene of an IC-free segment $\mathcal S$, i.e. $E'= E \cup \{ \{m^{ h }, m^{ t }\}~|~m \in \mathcal S\}$ (note that when $|\mathcal S |>1,~ w(\{m^{ h },m^{ t }\}) = 0$ since $\mathcal S$ is contiguous in all three extant genomes). In the following we refer to these edges as *conflict edges*. Let *C*(*m*) be the set of candidate median genes that are involved in an (external) conflict with a given candidate median gene *m* of $\mathcal S$, then the conflict edge $\{m^ h , m^ t \} \in E'$ is weighted by the maximum potential of a non-conflicting subset of *C*(*m*),

$$\begin{aligned} w'(\{m^ h , m^ t \}) = \max (\{\sum _{m'\in C'}\Delta (m')~|~C' \subseteq C(m) :~ C' \text { is conflict-free}\})\,. \end{aligned}$$

A conflict-free matching in $\Gamma '(\mathcal S)$ is a matching without a conflict edge.

##### **Lemma 1**

*Given an internal conflict-free segment* $\mathcal S$, *any maximum weight matching in graph* $\Gamma '(\mathcal S)$ *that is conflict-free defines a set of median genes and adjacencies that belong to at least one optimal FF-Median of the whole instance*.

##### *Proof*

Given an IC-free segment $\mathcal S= \{ m_1,\ldots ,m_k\}$ of an FF-Median instance $(G, H, I, \sigma )$. Let *M* be a conflict-free matching in graph $\Gamma '(\mathcal S)$. Because *M* is conflict-free and $\mathcal S$ contiguous in all three extant genomes, *M* must contain all candidate median genes of *S*. Now, let $M'$ be a median such that $\mathcal S \not \subseteq \mathcal C(M')$. Further, let *C*(*m*) be the set of candidate median genes that are involved in a conflict with with a given median gene *m* of $\mathcal S$ and $X = \mathcal C(M') \cap (\bigcup _{m \in \mathcal S} C(m) \cup \mathcal S)$. Clearly, $X \ne \emptyset$ and for the contribution  must hold , otherwise $M'$ is not optimal since it is straightforward to construct a median higher score which includes $\mathcal S$. Clearly, the contribution $\mathcal F(X)$ to the median is bounded by . But since $\mathcal S$ gives rise to a conflict-free matching with maximum score, also median $M''$ with $\mathcal C(M'') = (\mathcal C(M') \setminus X) \cup \mathcal C(\mathcal S)$ and $\mathcal A(M'') = (\mathcal A(M') \setminus \mathcal A(X)) \cup \mathcal A(S))$ must be an (optimal) median. $\square$

**Fig. 5:**
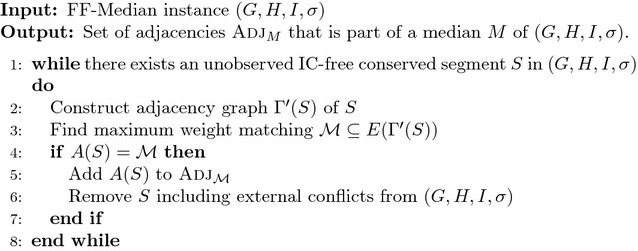
Algorithm ICF-SEG

Lemma 1 leads to a procedure (Fig. 5) that iteratively identifies and tests IC-free segments in the FF-Median instance. For each identified IC-free segment *S* an adjacency graph $\Gamma '(S)$ is constructed and a maximum weight matching is computed (Line 2–3). If the resulting matching is conflict-free (Line 4), adjacencies of IC-free segment *S* are reported and *S* is removed from an FF-Median instance by masking its internal adjacencies and removing all candidate median genes (and consequently their associated candidate median adjacencies) corresponding to external conflicts (Line 5–6). It then follows immediately from Lemma 1 that the set median genes returned by Fig. 5 belongs to at least one optimal solution to the FF-Median problem.

In the experiments, IC-free runs are used instead of segments. Step 1 is performed efficiently by first identifying maximal IC-free runs, then breaking it down into smaller runs whenever the condition in Step 4 is not satisfied.

### Solving problem FF-Adjacencies for three genomes

**Fig. 6:**
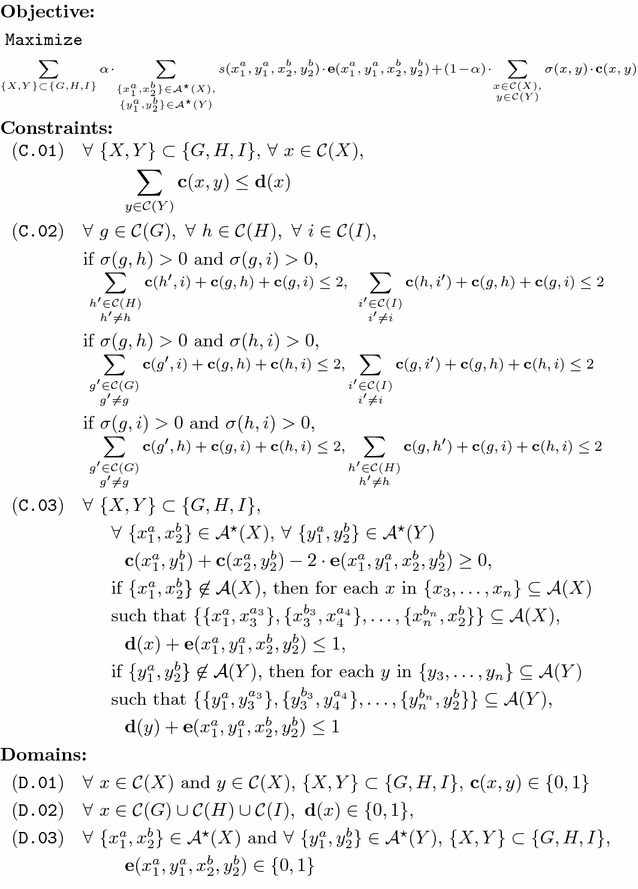
Program FFAdj-3G, an ILP for solving FF-Adjacencies for three genomes

We now describe program FFAdj-3G, as shown in Fig. 6. It returns an exact solution to problem FF-Adjacencies for three genomes *G*, *H*, and *I*, given their gene similarity graph $B = (G, H, I, E)$.

The objective of the integer linear program is to maximize a linear combination of the sum of adjacency scores of pairs of matched genes and the sum of similarities of matched genes. To evaluate the former sum, program FFadj-3G iterates over the sets of *candidate adjacences*, defined as $\mathcal A^\star (X) \equiv \cup _{X' \subseteq X} \mathcal A(X')$ over all subgenomes $X' \subseteq X$ of a given genome *X*.

FFAdj-3G makes use of three types of binary variables $\mathbf {c, d}$, and $\mathbf {e}$ (see domains (D.01) − (D.03)). Variables $\mathbf c(x, y)$ indicate if edge $\{x, y\}$ in gene similarity graph *B* is part of the anticipated matching $\mathcal M$. Likewise, each variable $\mathbf d(x)$, $x \in \mathcal C(G) \cup \mathcal C(H) \cup \mathcal C(I)$, encodes if vertex *x* in gene similarity graph *B* is potentially incident to an edge in $\mathcal M$. Lastly, variables $\mathbf e(x_1^a, y_1^a, x_2^b, y_2^b)$ indicate if gene extremities $x_1^a, x_2^b, y_1^a, y_2^b$, with $a, b \in \{\text {h, t}\}$ of the $\mathcal M$-induced subgenomes $X_{\mathcal M}$ and $Y_{\mathcal M}$ can possibly form conserved adjacencies, i.e., $\{x_1^a, x_2^b\} \in \mathcal A(X_{\mathcal M})$ and $\{y_1^a, y_2^b\} \in \mathcal A(Y_{\mathcal M})$.

**Fig. 7:**
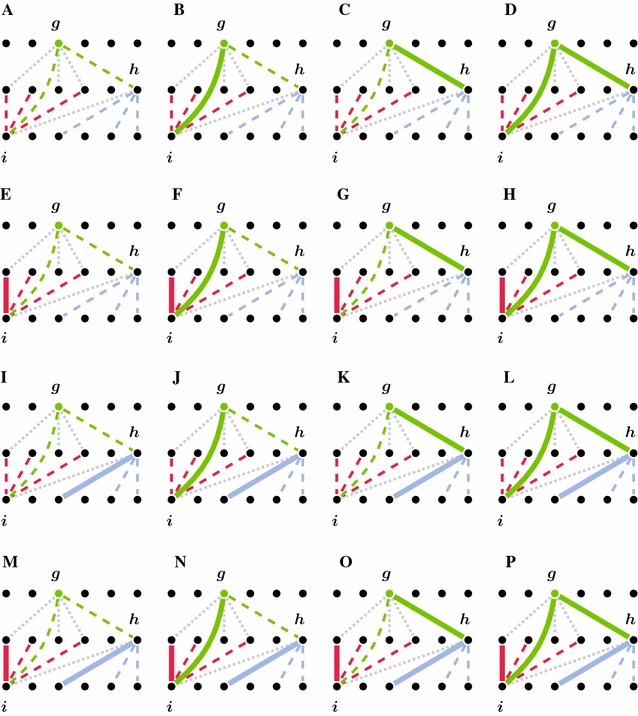
The implications of Constraint (C.02) on combinations of saturated edges. Parts **a**–**p** visualize all 16 possibilities that are valid under Constraint (C.01). The parts show how edges incident to genes *i* and *h* are effected by the first case of Constraint (C.02) that acts on edges $\{g, h\}$ and $\{g, i\}$ (*green lines*). Saturated edges are indicated by *thick continuous lines*, unsaturated edges by *dashed lines*. *Dotted gray lines* are not considered by the constraint and can be either saturated or unsaturated. Only combinations shown in Parts **h**, **l**, and **p** violate constraint (C.02)

Constraints (C.01) and (C.02) ensure that the resulting matching $\mathcal M$ forms a valid partial 3-matching. That is, no two genes of a connected component in the $\mathcal M$-induced subgraph of gene similarity graph *B* belong to the same genome (see Definition 1). In doing so, (C.01) establishes pairwise matching constraints, i.e., it guarantees that in the matching-induced subgraph, each gene is connected to at most one gene per genome. Note that variables $\mathbf d$ are assigned 1 for each gene that is incident to *at least one* edge of partial 3-matching $\mathcal M$. That is, the value of a variable $\mathbf b$ can be 1 even though its corresponding gene is not incident to an edge of $\mathcal M$. But then, program FFAdj-3G permits a gene to be incident to several edges of $\mathcal M$, if each of these edges is incident to genes of different genomes. Additional constraints are enforced by (C.02) on every pair of edges that share a common gene in one genome, but are incident to genes of different genomes. Let us consider three genes $g \in G, h \in H$, and $i \in I$, which are connected by two edges $\{g,h\}, \{g,i\} \in E$. This scenario is represented in Fig. 7, where the two edges $\{g,h\}$ and $\{g,i\}$ that share the common gene *g* are colored green. The figure schematizes all 16 combinations in which edges in the neighborhood of $\{g,h\}$ and $\{g,i\}$ (including $\{g,h\}$ and $\{g,i\}$) can participate in a matching only constrained by (C.01). Saturated edges are indicated by thick continuous lines, unsaturated edges by dashed lines, and gray dotted lines (which can be either saturated or unsaturated) are not considered by the two sum constraints. For instance, Fig. 7a represents the case in which no edge incident to vertices *g*, *h*, or *i* is saturated. When applying Constraint (C.02) on these 16 combinations, it is ensured that (i) the sum of saturated edges that are red or green is less than or equal to two, and (ii) that the sum of saturated edges that are blue or green is less than or equal to two. Combinations that violate any of the two sum constraints, shown in Fig. 7h, l, p, are exactly those that violate the partial 3-matching property. The gray dotted line between genes *h* and *i* indicates that edge $\{h,i\}$ is not considered by the constraints of (C.02). In case edge $\{h, i\}$ is saturated, it may be in conflict with saturated blue and red edges which results in violations of the pairwise matching constraints of (C.01).

Lastly, Constraint (C.03) covers the rules of forming conserved adjacencies: (i) it ensures that a variable $\mathbf e$, which indicates a conserved adjacency for two edges, is set to 1 only if the edges are saturated; (ii) using variables $\mathbf d$, it prohibits that no gene (and thus no incident edge) within a conserved adjacency is part of the matching.

## Experimental results and discussion

Our algorithms have been implemented in Python and require CPLEX^1^; they are freely available as part of the family-free genome comparison tool FFGC downloadable at http://bibiserv.cebitec.uni-bielefeld.de/ffgc.

In subsequent analyses, gene similarities are based on local alignment hits identified with BLASTP on protein sequences using an e-value threshold of $10^{-5}$. In gene similarity graphs, we discard spurious edges by applying a *stringency filter* proposed by Lechner et al. [13] that utilizes a local threshold parameter $f \in [0, 1]$ and BLAST bit-scores: a BLAST hit from a gene *g* to *h* is only retained if it is has a higher or equal score than *f* times the best BLAST hit from *h* to any gene $g'$ that is member of the same genome as *g*. In all our experiments, we set *f* to 0.5. Edge weights of the gene similarity graph are then calculated according to the *relative reciprocal BLAST score* (RRBS) [14]. Finally we applied algorithm ICF-SEG with conserved segments defined as runs.

For running programs FF-Median and FFAdj-3G, we granted CPLEX 64 CPU cores, 64 GB memory and a time limit of 1 h per dataset. In both simulated and real data we set the FFAdj-3G’s parameter $\alpha$ to 0.9.

In our experiments, we compare ourselves against the orthology prediction tool MultiMSOAR [11]. This tool requires precomputed gene families, which we constructed by following the workflow described in [11].

### Evaluation on simulated data

**Fig. 8:**
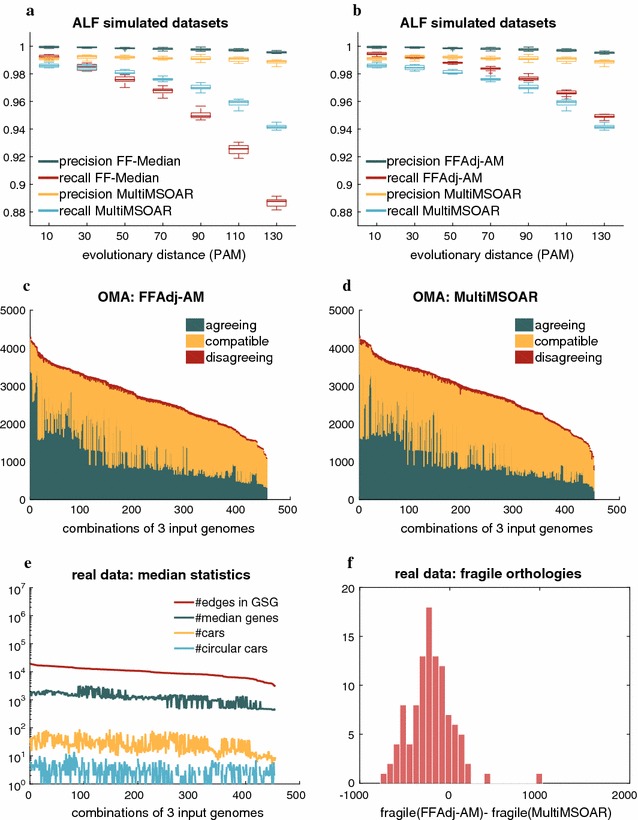
*Top* Precision and recall of **a** FF-Median and **b** FFAdj-AM in comparison with MultiMSOAR in simulations; *Middle* agreement, compatibility and disagreement of positional orthologs inferred by **c** FFAdj-AM and **d** MultiMSOAR with the OMA database; *Bottom* **e** statistical assessment of CARs and median genes inferred by FF-Median on real datasets; **f** histogram of fragile orthologies in results obtained by FFAdj-AM and MultiMSOAR

We first evaluate our algorithms on simulated data sets obtained by ALF [15]. The ALF simulator covers many aspects of genome evolution from point mutations to global modifications. The latter includes inversions and transpositions as genome rearrangement operations. Various options are available to customize the process of gene family evolution. In our simulations, we mainly use standard parameters suggested by the authors of ALF and we focus on three parameters that primarily influence the outcome of gene family-free genome analysis: (i) the rate of sequence evolution, (ii) the rate of genome rearrangements, and (iii) the rate of gene duplications and losses. We keep all three rates constant, only varying the evolutionary distance between the generated extant genomes. We confine our simulations to protein coding sequences. A comprehensive list of parameter settings used in our simulations is shown in Additional file 1: Table 2 of Section 2. As root genome in the simulations, we used the genomic sequence of an *Escherichia coli* K-12 strain (Accession No: NC_000913.2) which comprises 4320 protein coding genes. We then generated $7\times 10$ data sets with increasing evolutionary distance ranging from 10 to 130 *percent accepted mutations* (PAM). Details about the generated data sets are shown in Additional file 1: Table 1 of Section 2. Figure 8a, b show the outcome of our analysis with respect to precision and recall^2^ of inferring positional orthologs. In all simulations, program FF-Median and heuristic FFAdj-AM generated no or very few false positives, leading to perfect or near-perfect precision score, consistently outperforming MultiMSOAR. The comparison between orthologs inferred by FF-Median and FFAdj-AM shows that the additional orthologies identified by FFAdj-AM do not deteriorate the precision, but only improve its recall. Thus, our heuristic method consistently outperforms MultiMSOAR in precision and recall over all evolutionary distances.

#### Evaluation on real data

We study 15 $\gamma$-proteobacterial genomes that span a large taxonomic spectrum and are contained in the OMA database [16]. A complete list of species names is given in Additional file 1: Table 2 of Section 3. We obtained the genomic sequences from the NCBI database and constructed for each combination of three genomes a gene similarity graph following the same procedure as in the simulated dataset. In 9 out of the 455 combinations of genomes the time limit prohibited CPLEX from finding an optimal solution for program FF-Median. Likewise for FFAdj-AM, CPLEX was unable to find and optimal solution in 69 combinations within the provided 1h time frame. However, in all those cases CPLEX was still able to find integer feasible suboptimal solutions, many of which were less than a factor of 10% from the optimal. Figure 8e displays statistics of the medians constructed from the real dataset. The number of candidate median genes and adjacencies ranges from 756 to 18,005 and 3164 to 2,261,716, respectively, giving rise to up to 3223 median genes that are distributed on 5 to 90 CARs per median. Some CARs are circular, indicating dubious conformations mostly arising from tandem duplications, but the number of such cases were low (mean: 2.76, max: 14).

We observed that the gene families in the OMA database are clustered tightly and therefore missing many true orthologies in the considered triples of genomes. As a result, many of the orthologous groups inferred by FF-Median/FFAdj-AM and MultiMSOAR fall into more than one gene family inferred by OMA. We therefore evaluate our results by classifying the inferred orthologous groups into three categories: An orthologous group *agrees* with OMA if all its genes are in the same OMA group. It *disagrees* with OMA if any two of its genes *x* and *y* (of genomes *X* and *Y* respectively) are in different OMA groups but the OMA group of *x* contains another gene from genome *Y*. It is *compatible* with OMA if it neither agrees nor disagrees with OMA. We measure the number of orthologous groups of FFAdj-AM and MultiMSOAR in each of the three categories. Figure 8c, d give an overview on the outcome this analysis, showing that FFAdj-AM and MultiMSOAR perform roughly equally well.

The number of orthologous groups that disagree with OMA is comparably low for both FFAdj-AM (mean: 44.43, var: 129) and MultiMSOAR (mean: 44.63, var: 243). In total, FFAdj-AM is able to infer 7865 orthologies more that are agree and 94 less that disagree with OMA. Conversely, MultiMSOAR finds 69,769 more compatible orthologies than FFAdj-AM.

We then performed another analysis to assess the *fragility* of the positional orthology predictions. To this end, we look at orthologous groups across multiple datasets that share two extant genomes, but vary in the third. Given two genes, *x* of genome *X* and *y* of genome *Y*, an orthologous group that contains *x* and *y* is called *fragile* if *x* and *y* no longer occur not in the same orthologous group if the third extant genome is exchanged for another. We computed the total count of fragile orthologies produced by FFAdj-AM and MultiMSOAR for all 105 genome pairs in our dataset, see Fig. 8f. In 88 pairwise comparisons ($83.8\%$) the orthologous groups inferred by FFAdj-AM have fewer fragile orthologies than those by MultiMSOAR.

Overall, we can observe that FFAdj-AM performs equally well or better as MultiMSOAR—which is consistent with our observation on simulated data—while producing less fragile orthologies in general. This suggests FFAdj-AM is an interesting alternative to identify higher confidence positional orthologs.

## Conclusions and future work

Our main contributions in this work are (i) the introduction and analysis of a new problem, FF-Median, a generalization of the unconstrained breakpoint median of three, (ii) FFAdj-3G, an exact algorithm for solving problem FF-Adjacencies for three genomes, and (iii) FFAdj-AM, a heuristic method combining both programs FF-Median and FFAdj-3G. Our heuristic shows superior performance in simulations and comparable performance on real data compared to MultiMSOAR, a competing software tool.

One aim of future work is to investigate alternative methods to reduce the computational load of programs FF-Median and FFAdj-3G by identifying further strictly sub-optimal and optimal substructures, which might require a better understanding of the impact of internal conflicts within substructures defined by intervals in the extant genomes. Without the need to modify drastically either the FF-Median/FF-Adjacencies problem definition or the ILP, one can think about more complex weighting schemes for adjacencies that could account for known divergence time between genomes. With regard to program FF-Median, it would probably be interesting to combine this with the use of common intervals instead of runs to define conflict-free sub-instances.

## Additional file

**Additional file 1.** This file contains a hardness proof for problem FF-Median and additional information regarding the simulated and real dataset that were used in the evaluation of this study.

## Footnotes

1
http://www.ibm.com/software/integration/optimization/cplex-optimizer/.

2 Precision: #true positives/(#true positives + #false positives), recall: #true positives/(#true positives + #false negatives).
