# Supplementary material for: The gene family-free median of three
Source: Algorithms Mol Biol. 2017 May 26;12:14. doi: 10.1186/s13015-017-0106-z (PMC5446766; doi:10.1186/s13015-017-0106-z)
Supplement: Supplementary file 1 — Additional file 1. This file contains a hardness proof for problem FF-Median and additional information regarding the simulated and real dataset that were used in the evaluation of this study. [file 13015_2017_106_MOESM1_ESM.pdf]

Supporting Information
for
The gene family-free median of three

Daniel Doerr, Pedro Feijão, Metin Balaban, and Cedric Chauve

December 17, 2016

1 Hardness proof of Problem FF-Median

Theorem 1. *Problem FF-Median is MAX SNP-hard.*

Reduction. The *maximum independent set problem for graphs bounded by node degree* 3, denoted as MAX IS-3 is MAX SNP-hard [3]. The corresponding decision problem can be informally stated as follows: Given a graph Λ bounded by degree 3 and some number $l \geq 1$, does there exists a set of vertices $V' \subseteq V$ of size $|V'| = l$ whose induced subgraph is unconnected? In the following, we present a transformation scheme $\mathbf{R}$ to phrase Λ as FF-median instance $\mathbf{R}(\Lambda) = (G, H, I, \sigma)$ such that the value $\mathcal{F}_{\curlywedge}(M)$ of a median M of $\mathbf{R}(\Lambda)$ is limited by $\mathcal{F}_{\curlywedge}(M) \leq 2 \cdot l + 3$. In doing so, we associate vertices of V with genes of extant genomes G, H and I. In order to keep track of associated genes, we denote by function $\xi(x)$ the list of vertices associated with gene x. We further introduce two types of unassociated genes, "$\emptyset$" and "$*$", whose members are identified by subscript notation.

Transformation $\mathbf{R}$:

1. Construct genome G such that for each vertex $v \in V$ there exists two associated genes $g_v, \bar{g}_v \in \mathcal{C}(G)$, i.e. $\xi(g_v) = \xi(\bar{g}_v) = v$. Further, let each gene pair $g_v, \bar{g}_v$ form a circular chromosome, giving rise to adjacency set $\mathcal{A}(G) = \{\{\bar{g}_v^{\mathrm{h}}, g_v^{\mathrm{t}}\}, \{\bar{g}_v^{\mathrm{h}}, g_v^{\mathrm{t}}\} \mid v \in V,\ g_v, \bar{g}_v \in \mathcal{C}(G)\}$.

2. For each edge $(u, v) \in E$ construct a circular chromosome $\mathcal{X}_{uv}$ hosting two genes $x_{uv}, x_\emptyset \in \mathcal{C}(\mathcal{X}_{uv})$, with gene x_{uv} being associated with both vertices u and v and gene $x_\emptyset$ being unassociated. Further, let both genes form a circular chromosome, giving rise to adjacency set $\mathcal{A}(\mathcal{X}_{uv}) = \{\{x_{uv}^{\mathrm{h}}, x_\emptyset^{\mathrm{t}}\}, \{x_\emptyset^{\mathrm{h}}, x_{uv}^{\mathrm{t}}\}\}$.

3. Assign each chromosome constructed in the previous step either to genome H or to genome I such that each vertex $v \in V$ is associated with at most two genes per genome.

4. Complete genomes H and I with additional circular chromosomes $\mathcal{X}_v$ where $\mathcal{C}(\mathcal{X}_v) = \{x_v, x_\emptyset\}$ and $\mathcal{A}(\mathcal{X}_v) = \{\{x_v^{\mathrm{h}}, x_\emptyset^{\mathrm{t}}\}, \{x_\emptyset^{\mathrm{h}}, x_v^{\mathrm{t}}\}\}$ such that each vertex in V is associated with exactly two genes per genome.

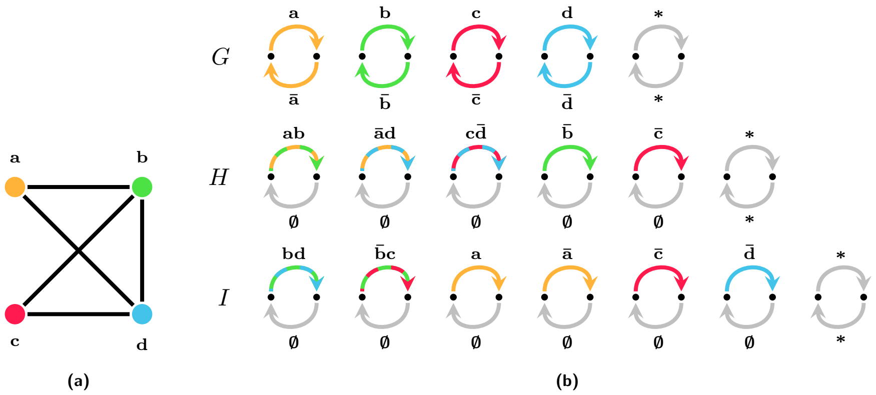

Figure 1: (a) A simple graph bounded by degree three and (b) a corresponding FF-Median instance constructed with transformation scheme **R**.

5. For each vertex $v \in V$, let $g, \bar{g} \in \mathcal{C}(G)$, $h, \bar{h} \in \mathcal{C}(H)$, and $i, \bar{i} \in \mathcal{C}(I)$ be the pairs of genes associated with v, i.e. $\xi(g) = \xi(\bar{g}) = \xi(h) \cap \xi(i) = \xi(\bar{h}) \cap \xi(\bar{i}) = v$. Assign gene similarities $\sigma(g, h) = \sigma(g, i) = \sigma(h, i) = 1$ and $\sigma(\bar{g}, \bar{h}) = \sigma(\bar{g}, \bar{i}) = \sigma(\bar{h}, \bar{i}) = 1$.

6. Add a copy of circular chromosome $\mathcal{X}_*$ to each genome G, H, and I, where $\mathcal{C}(\mathcal{X}_*) = \{x_*, \bar{x}_*\}$ and $\mathcal{A}(\mathcal{X}_*) = \{\{x_*^{\mathrm{h}}, \bar{x}_*^{\mathrm{t}}\}, \{\bar{x}_*^{\mathrm{h}}, x_*^{\mathrm{t}}\}\}$. Let $g_*, \bar{g}_* \in \mathcal{C}(G), h_*, \bar{h}_* \in \mathcal{C}(H)$, and $i_*, \bar{i}_* \in \mathcal{C}(I)$, set the gene similarity score between all pairs of genes in $\{g_*, h_*, i_*\}$ and $\{\bar{g}_*, \bar{h}_*, \bar{i}_*\}$ respectively, to 1. Lastly, set the gene similarity score of all pairs of unassociated genes of type "$\emptyset$" including genes $g_*, \bar{g}_*$ to $\frac{1}{4}$.

Except for step 3, none of the instructions of transformation scheme **R** are computationally challenging. Note that in step 3 the demanded partitioning of chromosomes into genomes H and I is always possible as consequence of Vizing's Theorem [4], by which every graph with maximum node degree d is edge-colorable using at most d or $d+1$ colors. Hence, using colors $\chi_1, \chi_2, \chi_3, \chi_4$ with $\chi_1 = \chi_2 \equiv I$, $\chi_3 = \chi_4 \equiv H$ and Misra and Gries' algorithm [2], edges of graph $\Lambda = (E, V)$ can be partitioned into two groups in $\mathcal{O}(|E||V|)$ time implying an assignment to genomes H and I.

Example 1. *Figure 1 (b) shows a FF-Median instance constructed with transformation scheme **R** from the simple graph depicted in Figure 1 (a). Gene similarities between genes are not shown, but can be derived from the genes' labeling.*

We structure our proof that the presented transformation is in fact a valid mapping of an MAX IS-3 instance to an instance of FF-Median into three different lemmas:

Lemma 1. *Given a median M of FF-Median instance $\mathbf{R}(\Lambda) = (G, H, I, \sigma)$, (1) for each median gene $(g, h, i) \in \mathcal{C}(M)$ where g, h, or i are associated with vertices in $V(\Lambda)$ holds $\xi(g) = \xi(h) \cap \xi(i) = v$, $v \in V(\Lambda)$; (2) there exist at most two median genes whose corresponding extant genes are not associated to any vertex in $V(\Lambda)$.*

Proof. Assume for contradiction that claim (1) does not hold. Then either $\xi(g) \neq \xi(h) \cap \xi(i)$, or $\xi(h) \cap \xi(i) = \emptyset$, both of which violate the constraint of establishing gene similarities between associated genes that is given in step 5. For claim (2), observe that the only unassociated genes in genome G are gene g_* and $\bar{g}_*$ introduced in step 6, limiting the overall number of unassociated genes in any median M. □

Lemma 2. *The conserved adjacency set of any median M of FF-Median instance $\mathbf{R}(\Lambda) = (G, H, I, \sigma)$ is of the form $\mathcal{A}(M) \cap \mathcal{A}^C_\curlywedge = \mathcal{A}^G_\curlywedge(M) \cup \{\{m^{\mathrm{h}}_*, \overline{m}^{\mathrm{t}}_*\}, \{\overline{m}^{\mathrm{h}}_*, m^{\mathrm{t}}_*\}\}$, where the extant genes corresponding to m_* and $\overline{m}_*$ are all unassociated genes of type "$*$" and $\mathcal{A}(M)^G_\curlywedge \subseteq \big\{\{m^{\mathrm{h}}_1, m^{\mathrm{t}}_2\} \in A^C_\curlywedge \mid \xi(\pi_G(m_1)) = \xi(\pi_G(m_2))\big\}$.*

Proof. Observe that both candidate median adjacencies $a_* = \{m^{\mathrm{h}}_*, \overline{m}^{\mathrm{t}}_*\}$ and $\bar{a}_* = \{\overline{m}^{\mathrm{h}}_*, m^{\mathrm{t}}_*\}$ are conserved in all three genomes, whereas all other conserved candidate adjacencies between associated and unassociated genes can be at most conserved in H and I. Establishing adjacencies $a_*, \bar{a}_*$ gives rise to a cumulative adjacency score of 6. Conversely, up to 4 non-conflicting adjacencies between associated and unassociated genes can be established that are conserved in both genomes H and I. However, since such adjacencies are only conserved between unassociated genes of type "$\emptyset$" whose gene similarities are set to $\frac{1}{4}$, the best cumulative adjacency score can not exceed 4. Thus, adjacencies $a_*, \bar{a}_*$ must be contained in any median. Further, because of this and the fact that in both genomes H and I, each gene associated with vertices of $V(\Lambda)$ is only adjacent to an unassociated gene, M cannot contain adjacencies that are conserved in extant genomes other than genome G, which are the adjacencies of each gene pair $(g_v, \bar{g}_v)$ associated with the same vertex $v \in V(\Lambda)$. □

Lemma 3. *Given FF-median instance $\mathbf{R}(\Lambda) = (G, H, I, \sigma)$, let m_u, m_v be any pair of candidate median adjacencies of $\mathcal{A}_\curlywedge$ whose corresponding extant genes are associated to vertices $u, v \in V(\Lambda)$, then m_u, m_v are conflicting if and only if $(u, v) \in E$.*

Proof. By construction in step 5 of transformation scheme $\mathbf{R}$, each vertex $v \in V$ is associated with exactly two candidate median genes $m_v = (g, h, i), \overline{m}_v = (\bar{g}, \bar{h}, \bar{i})$, $m_v, \overline{m}_v \in \Sigma_\curlywedge$, such that $\xi(g) = \xi(h) \cap \xi(i) = v$ and $\xi(\bar{g}) = \xi(\bar{h}) \cap \xi(\bar{i}) = v$. Further, let u be another vertex of $V(\Lambda)$, such that $(u, v) \in E(\Lambda)$, and $m_u, \overline{m}_u$ are its two corresponding candidate median genes. Then, by construction in step 2, there exists exactly one extant gene x with $\xi(x) = uv$ (which, by assignment in step 3, is either contained in genome H or I). Consequently, either m_u is in conflict with m_v, or $\overline{m}_u$ with $\overline{m}_v$, or $\overline{m}_u$ with m_v, or m_u with $\overline{m}_v$. Recall that by construction in step 2 in $\mathbf{R}$ and by Lemma 2, $m_u, \overline{m}_u$ and $m_v, \overline{m}_v$ form conserved candidate adjacencies $\{m^{\mathrm{h}}_u, \overline{m}^{\mathrm{t}}_u\}$, $\{\overline{m}^{\mathrm{h}}_u, m^{\mathrm{t}}_u\}$ and $\{m^{\mathrm{h}}_v, \overline{m}^{\mathrm{t}}_v\}$, $\{\overline{m}^{\mathrm{h}}_v, m^{\mathrm{t}}_v\}$, respectively. Clearly, independent of which of the candidate median gene pairs of u and v are in conflict, both pairs of candidate median adjacencies are in conflict with each other.

Now, let u, v be two vertices of $V(\Lambda)$ such that edge $(u, v) \notin E(\Lambda)$, then there exists no gene x in extant genomes H and I with $\xi(x) = uv$. Even more, due to Lemma 1, there cannot exist a candidate median gene (g, h, i) with $\{u, v\} \subseteq \xi(g) \cup \xi(h) \cup \xi(i)$. Thus, the candidate median genes of u and v are not conflicting and neither are their corresponding candidate median adjacencies. □

We proceed to show that the given transformation scheme gives rise to an approximation preserving reduction known as *L-reduction*. An L-reduction reduces a problem P to a problem Q by means of two polynomial-time computable transformation functions: A function $f : P \to Q' \subseteq Q$ that maps each instance of P onto an instance of Q, herein represented by transformation scheme $\mathbf{R}$, and a function $g : Q' \to P$ to transform any feasible solution of an instance in Q' to a feasible solution of an instance of P. Here, a *feasible* solution means any – not necessarily *optimal* – solution that obeys the problem's constraints. A feasible solution of FF-Median instance (G, H, I, σ) is an *ancestral genome* X where $\mathcal{C}(X) \subseteq \Sigma_\curlywedge$ and $\mathcal{A}(X) \subseteq \mathcal{A}_\curlywedge$ such that $\mathcal{A}(X)$ is conflict-free. We give the following transformation scheme to map ancestral genomes of an FF-Median instance to solutions of an MAX IS-3 instance:

Transformation $\mathbf{S}$: Given any ancestral genome X of $\mathbf{R}(\Lambda)$, return $\{\xi(\pi_G(m_1)) \mid \{m_1^a, m_2^b\} \in \mathcal{A}(X) : \mathbb{I}_G(\pi_G(m_1)^a, \pi_G(m_2)^b) = 1 \text{ and } \xi(\pi_G(m_1)) \neq \emptyset\}$.

We define score function $s_\curlywedge(X) \equiv \frac{1}{2}\mathcal{F}_\curlywedge(X) - 3$ of an ancestral genome X. For $(\mathbf{R}, \mathbf{S})$ to be an L-reduction the following two properties must hold for any given MAX IS-3 instance (Λ, l): (1) There is some constant α such that for any median M of the transformed FF-Median instance $\mathbf{R}(\Lambda)$ holds $s_\curlywedge(M) \leq \alpha \cdot l$; (2) There is some constant β such that for any ancestral genome X of $\mathbf{R}(\Lambda)$ holds $l - |\mathbf{S}(X)| \leq \beta \cdot |s_\curlywedge(M) - s_\curlywedge(X)|$. We proceed to proof the following lemma:

Lemma 4. $(\mathbf{R}, \mathbf{S})$ *is an L-reduction of problem MAX IS-3 to problem FF-Median with* $\alpha = \beta = 1$.

Proof. For any median M of FF-Median instance $\mathbf{R}(\Lambda)$, the number of conserved median adjacencies with correspondence to the same vertex of Λ is two, giving rise a cumulative adjacency score of two. From Lemmata 2 and 3 immediately follows that any ancestral genome of $\mathbf{R}(\Lambda)$ that maximizes the number of conserved adjacencies also maximizes the number of independent vertices in Λ. Recall that the two conserved adjacencies between unassociated genes of type "$*$" (which are part of all medians) give rise to a cumulative adjacency score of 6, we conclude that $|\mathcal{A}(M) \cap \mathcal{A}_\curlywedge^C| - 2 = \frac{1}{2}\mathcal{F}_\curlywedge(M) - 3 = s_\curlywedge(M) = l$, thus $\alpha = 1$.

Because $l = s_\lambda(M)$, it remains to show that $l - |S(X)| \leq \beta|l - s_\curlywedge(X)|$. In a *sub-optimal* ancestral genome of $\mathbf{R}(\Lambda)$, median genes with no association to vertices of Λ can also contain extant genes of type "$\emptyset$". These unassociated median genes can form "mixed" conserved adjacencies with genes that are associated with vertices of Λ. Such mixed conserved adjacencies have no correspondence to vertices in Λ and do not contribute to the transformed solution $\mathbf{S}(X)$ of an ancestral genome X. Yet, as mentioned earlier, the cumulative adjacency score of all mixed conserved adjacencies can not not exceed 4. Therefore it holds that $|S(X)| \geq s_\curlywedge(X)$ and we conclude $\beta = 1$. □

2 Simulated sequence evolution with ALF

PAM	Genome	Inversions	Transpositions	Duplications	Losses
	G	8.7	6.1	7.3	6.9
10	H	7.3	4.5	6.3	5.4
	I	8.5	6.6	10.4	5.6
	G	24.5	16.9	21.0	22.7
30	H	23.4	19.8	20.6	18.4
	I	25.5	17.2	17.5	20.9
	G	39.9	27.8	32.4	36.7
50	H	41.8	31.8	31.0	31.7
	I	43.2	30.0	28.7	39.7
	G	58.6	42.3	41.1	39.2
70	H	57.0	43.6	46.3	45.1
	I	60.4	41.4	40.7	39.1
	G	75.0	54.5	53.1	64.2
90	H	69.9	50.5	54.1	65.0
	I	75.2	55.5	60.3	58.5
	G	96.3	69.4	67.0	74.6
110	H	90.6	64.2	62.5	70.9
	I	90.2	68.5	62.6	61.2
	G	105.7	76.3	74.4	81.0
130	H	108.7	78.2	79.6	82.8
	I	110.8	73.6	73.9	77.3

Table 1: Average benchmark data of seven evolutionary distances, each comprising ten genomic datasets generated by ALF [1].

Parameter name	Value	
sequence evolution		
substitution model	WAG (amino acid substitution model)	
insertion and deletion	Zipfian distribution	exponent $c = 1.8214$
	insertion rate	0.0003
	maximum insertion length	50
rate variation among sites	Γ-distribution	shape parameter $a = 1$
	number of classes	5
	rate of invariable sites	0.01
genome rearrangement		
inversion	rate	0.0004
	maximum inversion length	100
transposition	rate	0.0002
	maximum transposition length	100
	rate of inverted transposition	0.1
gene family evolution		
gene duplication	rate	0.0001
	max. no. of genes involved in one dupl.	5
	probability of transposition after dupl.	0.5
	fission/fusion after duplication	0.1
	probability of rate change	0.2
	rate change factor	0.9
	probability of temporary rate change (duplicate)	0.5
	temporary rate change factor (duplicate)	1.5
	life of rate change (duplicate)	10 PAM
	probability of temporary rate change (orig+duplicate)	0.3
	temporary rate change factor (orig+duplicate)	1.2
	life of rate change (orig+duplicate)	10 PAM
gene loss	rate	0.0001
	maximum length of gene loss	5
gene fission/fusion	rate	0.0
	maximum number of fused genes	−

Table 2: Parameter settings for simulations generated by ALF [1].

3 Real genomes dataset

Genbank ID	Name
U00096.3	Escherichia coli str. K-12 substr. MG1655, complete genome
AE004439.1	Pasteurella multocida subsp. multocida str. Pm70, complete genome
AE016853.1	Pseudomonas syringae pv. tomato str. DC3000, complete genome
AM039952.1	Xanthomonas campestris pv. vesicatoria complete genome
CP000266.1	Shigella flexneri 5 str. 8401, complete genome
CP000305.1	Yersinia pestis Nepal516, complete genome
CP000569.1	Actinobacillus pleuropneumoniae L20 serotype 5b complete genome
CP000744.1	Pseudomonas aeruginosa PA7, complete genome
CP000766.3	Rickettsia rickettsii str. Iowa, complete genome
CP000950.1	Yersinia pseudotuberculosis YPIII, complete genome
CP001120.1	Salmonella enterica subsp. enterica serovar Heidelberg str. SL476, complete genome
CP001172.1	Acinetobacter baumannii AB307-0294, complete genome
CP001363.1	Salmonella enterica subsp. enterica serovar Typhimurium str. 14028S, complete genome
FM180568.1	Escherichia coli 0127:H6 E2348/69 complete genome, strain E2348/69
CP002086.1	Nitrosococcus watsoni C-113, complete genome

Table 3: Dataset of genomes used in comparison with the OMA database.

References

[1] D A Dalquen, M Anisimova, G H Gonnet, and C Dessimoz. Alf – a simulation framework for genome evolution. *Mol. Biol. Evol.*, 29(4):1115–1123, 2012.

[2] J Misra and D Gries. A constructive proof of Vizing's theorem. *Inform. Process. Lett.*, 41(3):131–133, 1992.

[3] C H Papadimitriou and M Yannakakis. Optimization, approximation, and complexity classes. *J. Comp. Sys. Sci.*, 43(3):425–440, 1991.

[4] V G Vizing. On an estimate of the chromatic class of a p-graph. *Diskret. Analiz No.*, 3:25–30, 1964.
